# Colorectal cancer patients with different C-reactive protein levels and 5-year survival times can be differentiated with quantitative serum proteomics

**DOI:** 10.1371/journal.pone.0195354

**Published:** 2018-04-09

**Authors:** Matilda Holm, Mayank Saraswat, Sakari Joenväärä, Ari Ristimäki, Caj Haglund, Risto Renkonen

**Affiliations:** 1 Department of Pathology, University of Helsinki and Helsinki University Hospital, Helsinki, Finland; 2 Department of Surgery, University of Helsinki and Helsinki University Hospital, Helsinki, Finland; 3 Genome-Scale Biology Research Program, Research Programs Unit, University of Helsinki, Helsinki, Finland; 4 Translational Cancer Biology Research Program, Research Programs Unit, University of Helsinki, Helsinki, Finland; 5 Transplantation Laboratory, Haartman Institute, University of Helsinki, Helsinki, Finland; 6 HUSLAB, Helsinki University Hospital, Helsinki, Finland; University of South Alabama Mitchell Cancer Institute, UNITED STATES

## Abstract

Over 1.4 million people are diagnosed with colorectal cancer (CRC) each year, making it the third most common cancer in the world. Increased screening and therapeutic modalities including improved combination treatments have reduced CRC mortality, although incidence and mortality rates are still increasing in some areas. Serum-based biomarkers are mainly used for follow-up of cancer, and are ideal due to the ease and minimally invasive nature of sample collection. Unfortunately, CEA and other serum markers have too low sensitivity for screening and preoperative diagnostic purposes. Increasing interest is focused on the possible use of biomarkers for predicting treatment response and prognosis in cancer. In this study, we have performed mass spectrometry analysis (UPLC-UDMS^E^) of serum samples from 19 CRC patients. Increased levels of C-reactive protein (CRP), which occur during local inflammation and the presence of a systemic inflammatory response, have been linked to poor prognosis in CRC patients. We chose to analyze samples according to CRP values by dividing them into the categories CRP <30 and >30, and, separately, according to short and long 5-year survival. The aim was to discover differentially expressed proteins associated with poor prognosis and shorter survival. We quantified 256 proteins and performed detailed statistical analyses and pathway analysis. We discovered multiple proteins that are up- or downregulated in patients with CRP >30 as compared to CRP <30 and in patients with short as compared to long 5-year survival. Pathways that were enriched include LXR/RXR activation, FXR/RXR activation, complement and coagulation cascades and acute phase signaling response, with some of the proteins we identified having roles in these pathways. In this study, we have identified multiple proteins, of which a few have been previously identified as potential biomarkers, and others that have been identified as potential biomarkers for CRC for the first time, to the best of our knowledge. While these proteins still need to be validated in larger patient series, this pilot study will pave the way for future studies aiming to provide better biomarkers for patients with CRC.

## Introduction

Colorectal cancer (CRC) is the third most common cancer worldwide, accounting for almost 10% of the global cancer burden with over 1.4 million new cases and 700 000 deaths each year. While the 5-year survival rate for CRC is around 60%, only 40% of CRC patients are diagnosed when the disease occurs locally [1, 2]. This is partly due to limited resources in many countries as well as a lack of widespread screening programs [3]. Even for patients who undergo curative resection the prognosis is quite poor, with only 50% of patients surviving 5 years [4]. Progress has been made to reduce CRC incidence and mortality over the past decade through increased screening and improved treatments such as the development of new combination therapies [2, 5]. For example, the addition of either bevacizumab or panitumumab to chemotherapy consisting of oxaliplatin, fluorouracil and leucovorin (FOLFOX4) has contributed to better survival in CRC patients [6, 7]. However, CRC still remains a major cause of cancer death.

Biomarkers are molecules that change upon transition to a pathological state and can be used to indicate the presence of disease. They can be used for screening and detection of cancer, as well as for monitoring of treatment and during follow-up. While the TNM staging system provides a standard basis for staging and a prediction of survival, biomarkers can provide information concerning the subdivision of tumor classes into subgroups that exhibit different behavior [8, 9]. Proteins detectable in serum are routinely used as biomarkers, such as carcinoembryonic antigen (CEA), which is the most widely used biomarker for CRC. However, CEA is not useful for the detection of early CRC due to lack of sensitivity and specificity, which limits its usefulness [10, 11].

Proteomics is the study of the complete proteome of a biological sample, and proteomic techniques such as mass spectrometry are widely used to search for biomarkers. Due to the need for new biomarkers with better sensitivity and specificity, searches to identify new potential proteins that could serve as biomarkers with clinical use are ongoing. Through proteomic analysis several serum proteins with diagnostic potential for CRC have already been identified, and there are likely more awaiting discovery [10, 12]. In our study, we decided to analyze serum samples from CRC patients according to C-reactive protein (CRP) values and, separately, according to 5-year survival. Increased levels of CRP, an acute-phase plasma protein whose concentration increases during both local inflammation and during a systemic inflammatory response (SIR), have been linked to poorer survival in CRC patients [13, 14]. Therefore, it is of interest to identify proteins that are differentially expressed in patients with high CRP values and a concomitant poorer prognosis, as well as in patients with short 5-year survival. Identifying patients with a poor prognosis would help select those who would benefit from more aggressive treatment and assist patients with a good prognosis in avoiding unnecessarily harsh treatment.

In this pilot study, we have used Ultra Performance Liquid Chromatography-Ultra Definition Mass Spectrometry (UPLC-UDMS^E^)-based proteomics to compare serum samples from 19 CRC patients. The samples were first analyzed after dividing them into two categories based on CRP, CRP <30 and CRP >30, and the same samples were analyzed again after being divided into the categories short and long 5-year survival. In this study, we have quantified 256 proteins with two or more unique peptides. Data were further analyzed by ANOVA, principal component analysis (PCA), Orthogonal Projections to Latent Structures Discriminant Analysis (OPLS-DA)-modeling and OPLS-DA-associated S-plot. Pathway analysis was performed using Integrated Molecular Pathway Level Analysis (IMPaLa) and Ingenuity Pathway Analysis (IPA). In our pilot study, we propose several potential biomarkers, which have good statistical significance.

## Material and methods

### Patient samples

The study included preoperative serum samples from 19 patients with colon cancer who underwent hemicolectomy resection with curative intent at Department of Surgery, Helsinki University Hospital between September 1999 and June 2006. Eight patients had an elevated CRP value, without any signs or symptoms of infection, but were considered to have a systemic inflammatory response due to their cancer. Eleven patients had an extremely low CRP value of 0. The patient details are given in S1 Table. The CRP values were measured according to the routine method of the clinical laboratory, Helsinki University Hospital. The clinical data came from patient records, the survival data from Population Register Centre of Finland and the cause of death for all those deceased from Statistics Finland. The study was approved by the Surgical Ethics Committee of Helsinki University Hospital (Dnro HUS 226/E6/06, extension TMK02 §66 17.4.2013). A written informed consent was obtained from all the participants in this study.

### Serum sample processing and trypsin digestion

Serum samples were processed essentially as previously described [15] and the protocol was repeated here. Briefly, serum samples were thawed and TOP 12 proteins were depleted using the TOP12 protein depletion kit (Pierce, ThermoFisher) according to the manufacturer’s instructions. Total protein concentration was estimated with Pierce BCA assay kit (Pierce, ThermoFisher). Total proteins (100 μg) from TOP12 depleted serum were aliquoted and dried by speedvac (Savant, ThermoFisher). Dried proteins were dissolved in 35 μL of 50 mmol/L Tris buffer, pH 7.8 containing 6M urea. Further, 1.8 μL of 200 mmol/L DTT was added to the samples and mixture was incubated at RT for 1 h with shaking. Iodoacetamide (7 μL of 200 mmol/L stock solution) was added to the total protein mixture with shaking at RT for 1 h. To quench excess iodoacetamide, DTT (7 μL of 200 mmol/L) was added to protein samples with shaking for 1 h at RT. After diluting the samples with 270 μL of MQ water, trypsin was added at 1:50 trypsin:protein ratio and protein mixture was digested at 37°C overnight. 30 μg of tryptic peptides were cleaned with C18 spin columns (Pierce, ThermoFisher). Cleaned peptides were dissolved to reach final concentration of 1.4μg/4μL in 0.1% formic acid. 12.5 fmol/μL of Hi3 spike-in standard peptides (Waters, MA, USA) were added to facilitate quantification.

### Liquid chromatography-mass spectrometry (LC-MS) and quantification

#### UPLC-MS

UPLC-MS was performed as described previously [15]. Briefly, four μL samples (equivalent to ~1.4 μg total protein) were injected to nano Acquity UPLC (Ultra Performance Liquid Chromatography)–system (Waters Corporation, MA, USA). TRIZAIC nanoTile 85 μm × 100 mm HSS-T3u wTRAP was used as separation device. Samples were loaded, trapped and washed for two minutes with 8.0 μL/min with 1% B. The analytical gradient used is as follows: 0–1 min 1% B, at 2 min 5% B, at 65 min 30% B, at 78 min 50% B, at 80 min 85% B, at 83 min 85% B, at 84 min 1% B and at 90 min 1% B with 450 nL/min. Buffer A was 0.1% formic acid in water and buffer B was 0.1% formic acid in acetonitrile. Data were acquired using HDMSE mode with Synapt G2-S HDMS (Waters Corporation, MA, USA). Data was collected in the range of 100–2000 m/z, scan time one-second, IMS wave velocity 650 m/s. Collision energy was ramped from 20 to 60 V. Calibration was performed with Glu1-Fibrinopeptide B MS2 fragments. Glu1-Fibrinopeptide B precursor ion was used as a lock mass during the runs. The samples were run in triplicates.

#### Data analysis

Data analysis [15, 16] and label-free quantification [17] were performed as previously described. The information will be repeated here. Briefly, the raw files were imported to Progenesis QI for proteomics software (Nonlinear Dynamics, Newcastle, UK). Lock mass of 785.8426 m/z, (doubly charged Glu1-Fibrinopeptide B) was used for mass correction. Peak picking and alignment were performed with default parameters of the algorithm. The peptide identification was done against Uniprot human FASTA sequences (UniprotKB Release 2015_09, 20205 sequence entries) which included ClpB protein sequence (CLPB_ECOLI (P63285)), which was inserted for label-free quantification. Fixed modification at cysteine (carbamidomethyl) and variable at methionine (oxidation) were used. Trypsin was specified as digesting enzyme with one missed cleavage allowed. False discovery rate (FDR) was set to less than 4% and auto error tolerances for fragment and precursor were used. Minimum one ion fragments per peptide, minimum three fragments per protein and minimum one peptide per protein were marked as “required” for ion matching.

Parsimony principle was used to group the proteins however, peptides unique to the proteins are also given as output. According to the parsimony principle, protein hits are reported as the minimum set comprising of all observed peptides. However, Progenesis QI for proteomics does not follow a strict parsimonious approach because of over-stringency [18]. However, to resolve conflicts, if two proteins are found with common peptides, protein with fewer peptides is immersed into the protein with more number of peptides. All relevant proteins are given in the output as a group under the lead protein having highest coverage or the highest score if the coverages of proteins are equal. Lead identity peptide data is used for quantitation and further details of this approach are given on the company website (www.nonlinear.com). The ANOVA relies on assumption that the conditions are independent and the means of the conditions are equal. Every peptide injection also contained 50 fmol of six CLPB_ECOLI (P63285, ClpB protein) peptides (Hi3 E. coli Standard, Waters). Peptide abundances were normalized with Hi3 spiked standard and relative quantitation was done with the non-conflicting peptides found. Signal of the protein is the average of the abundances of the comprised peptides. Details of the Progenesis software can be found on the software website (www.nonlinear.com) and in published literature [19]. Protein-wise differences between controls and cases were tested with ANOVA. Progenesis QI for proteomics was used for performing principle component analysis. The mass spectrometry proteomics data have been deposited to the ProteomeXchange Consortium via the PRIDE [20] partner repository with the dataset identifier PXD008583.

## Results

### Metadata

Serum samples from 19 CRC patients were analyzed in this study. The patients’ age ranged from 41 to 95 years old. CRP values ranged from 0–196 mg/L and were subsequently divided into the categories CRP <30 or >30 mg/L, based on a previous study [14]. Categories for survival were determined as long (alive five years post-surgery) or short (deceased within five years post-surgery) 5-year survival.

### Proteomic analysis

In this study we analyzed CRC samples by UPLC-UDMSE and quantified 387 proteins from serum with a minimum of one unique peptide. When filtered to proteins with two or more unique peptides, this number was reduced to 256 proteins, which were subsequently used for analysis (S2 Table). Confidence score of identification ranged from 3733,5 to 9,1 and fold changes ranged from 1063,3 to 1,4.

### CRP dataset

Altogether, 45 proteins passed the cut-off of ANOVA p-value 0.05 when the highest mean was set to CRP <30 and 26 proteins when the highest mean was set to CRP >30. The first 20 proteins to pass the cut-off are presented in Table 1, and all 71 proteins are listed in S3 Table. Our main criterion for identifying proteins different between the classes of samples was ANOVA p-values and therefore, proteins with p-values greater than 0.05 were not considered to be different.

**Table 1:** The first 20 proteins with 2 or more unique peptides analyzed according to CRP values and passing the cut-off of 0.05 for ANOVA. Accession, peptide count, unique peptides, confidence score, ANOVA p-value, maximum fold change and highest and lowest mean condition as well as the full protein name are given in the table.

Accession	Peptide count	Unique peptides	Confidence score	Anova (p)	Max fold change	Highest mean condition	Lowest mean condition	Description
P18065	5	5	33.0	0.000063	2.41	CRP >30	CRP < 30	Insulin-like growth factor-binding protein 2 OS = Homo sapiens GN = IGFBP2 PE = 1 SV = 2
P35372	2	2	11.3	8.94E-05	6.74	CRP >30	CRP < 30	Mu-type opioid receptor OS = Homo sapiens GN = OPRM1 PE = 1 SV = 2
P02741	3	2	29.3	0.0001	2.85	CRP >30	CRP < 30	C-reactive protein OS = Homo sapiens GN = CRP PE = 1 SV = 1
P0DJI8	30	19	189.2	0.0003	5.22	CRP >30	CRP < 30	Serum amyloid A-1 protein OS = Homo sapiens GN = SAA1 PE = 1 SV = 1
Q96PD5	51	46	510.5	0.0006	1.54	CRP < 30	CRP >30	N-acetylmuramoyl-L-alanine amidase OS = Homo sapiens GN = PGLYRP2 PE = 1 SV = 1
P02655	9	8	116.2	0.0006	3.43	CRP < 30	CRP >30	Apolipoprotein C-II OS = Homo sapiens GN = APOC2 PE = 1 SV = 1
Q8WVJ2	3	3	30.1	0.0011	2.99	CRP < 30	CRP >30	NudC domain-containing protein 2 OS = Homo sapiens GN = NUDCD2 PE = 1 SV = 1
P08887	2	2	9.6	0.0013	4.48	CRP >30	CRP < 30	Interleukin-6 receptor subunit alpha OS = Homo sapiens GN = IL6R PE = 1 SV = 1
Q9Y5I1	2	2	16.0	0.0014	1.56	CRP >30	CRP < 30	Protocadherin alpha-11 OS = Homo sapiens GN = PCDHA11 PE = 2 SV = 1
P78380	2	2	9.3	0.0015	4.03	CRP >30	CRP < 30	Oxidized low-density lipoprotein receptor 1 OS = Homo sapiens GN = OLR1 PE = 1 SV = 1
Q9Y6K1	5	3	40.8	0.0017	1.35	CRP < 30	CRP >30	DNA (cytosine-5)-methyltransferase 3A OS = Homo sapiens GN = DNMT3A PE = 1 SV = 4
P42081	3	2	15.3	0.0023	1.66	CRP < 30	CRP >30	T-lymphocyte activation antigen CD86 OS = Homo sapiens GN = CD86 PE = 1 SV = 2
Q6ZSA8	2	2	16.4	0.0029	3.53	CRP >30	CRP < 30	Putative uncharacterized protein FLJ45684 OS = Homo sapiens PE = 5 SV = 2
P07225	61	49	549.2	0.0030	1.28	CRP >30	CRP < 30	Vitamin K-dependent protein S OS = Homo sapiens GN = PROS1 PE = 1 SV = 1
P37088	5	5	39.7	0.0033	3.29	CRP >30	CRP < 30	Amiloride-sensitive sodium channel subunit alpha OS = Homo sapiens GN = SCNN1A PE = 1 SV = 1
P04180	9	9	52.5	0.0038	1.45	CRP < 30	CRP >30	Phosphatidylcholine-sterol acyltransferase OS = Homo sapiens GN = LCAT PE = 1 SV = 1
P00734	103	78	785.3	0.0039	1.24	CRP < 30	CRP >30	Prothrombin OS = Homo sapiens GN = F2 PE = 1 SV = 2
P19823	115	97	858.1	0.0041	1.59	CRP < 30	CRP >30	Inter-alpha-trypsin inhibitor heavy chain H2 OS = Homo sapiens GN = ITIH2 PE = 1 SV = 2
P08294	3	3	21.8	0.0041	2.68	CRP < 30	CRP >30	Extracellular superoxide dismutase [Cu-Zn] OS = Homo sapiens GN = SOD3 PE = 1 SV = 2
P15169	26	21	248.7	0.0044	1.62	CRP >30	CRP < 30	Carboxypeptidase N catalytic chain OS = Homo sapiens GN = CPN1 PE = 1 SV = 1

#### Principal component analysis (PCA)

**Fig 1:**
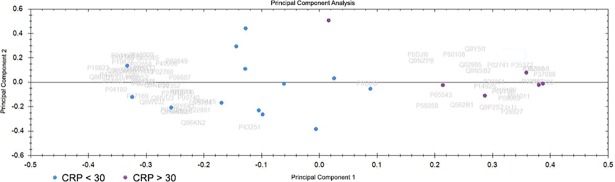
Principal component analysis (PCA). Blue dots are CRC samples with CRP <30 and purple dots are samples with CRP >30. The PCA was performed on proteins with 2 or more unique peptides that passed the cut-off of 0.05 for ANOVA.

PCA was performed using the software Progenesis QI for Proteomics. It determines the main axes of variations in a dataset and points out outliers. The PCA biplot helps to establish the differences between two or more classes of samples and visualize them. The PCA biplot of CRP <30 and >30 samples is shown in Fig 1. Only proteins with 2 or more unique peptides and an ANOVA p-value of less than 0.05 were considered for this PCA. A separation between the two groups can be seen here.

#### Orthogonal Projections to Latent Structures Discriminant Analysis (OPLS-DA)

OPLS-DA is a modeling technique used to model the data from two classes in order to present the differences between these classes. Only proteins with 2 or more unique peptides and an ANOVA p-value of less than 0.05 were used for OPLS-DA modeling. From this model, an S-plot can be generated, where the x-axis is the measure of change in a particular analyte and the y-axis is the significance of the analyte in the comparison between two groups. The S-plot can be used to find the proteins that are most significantly different in the two groups. An S-plot was generated for the proteins found in the analysis of the CRP <30 and >30 categories. Proteins passing the cut-off value of +0.7 or -0.7 for p(corr) values were considered to be significantly different and are presented in Table 2. Insulin-like growth factor-binding protein 2 (IGFBP2), Protocadherin alpha-11 (PCDHA11), Mu-type opioid receptor (OPRM1) and Serum amyloid A-1 protein (SAA1) were found to be upregulated in the CRP >30 category. Apolipoprotein C-II (APOC2) and N-acetylmuramoyl-L-alanine amidase (PGLYRP2) were found to be downregulated in the CRP >30 category.

**Table 2:** Proteins significantly different in the S-plot generated by comparing CRP <30 and >30 categories. Peptide count is the total number of peptides found for the given protein and unique peptides are the number of peptides unique to that protein out of the total peptides. Confidence score, ANOVA p-value, highest and lowest mean condition, the full name of the protein, covariance (p[1]) and correlation (p(corr)[1]) are shown in the table.

Primary accession	Peptide count	Unique peptides	Confidence score	Anova (p)	Max fold change	Highest mean condition	Lowest mean condition	Description	p[1]	p(corr)[1]
P18065	5	5	33.0439	6.31E-05	2.4114	CRP >30	CRP < 30	Insulin-like growth factor-binding protein 2 OS = Homo sapiens GN = IGFBP2 PE = 1 SV = 2	0.189875	0.875512
Q9Y5I1	2	2	15.9705	0.001471215	1.5643	CRP >30	CRP < 30	Protocadherin alpha-11 OS = Homo sapiens GN = PCDHA11 PE = 2 SV = 1	0.0294086	0.75569
Q6ZSA8	2	2	16.4398	0.002917281	3.5307	CRP >30	CRP < 30	Putative uncharacterized protein FLJ45684 OS = Homo sapiens PE = 5 SV = 2	0.105306	0.735447
P35372	2	2	11.3036	8.94E-05	6.7394	CRP >30	CRP < 30	Mu-type opioid receptor OS = Homo sapiens GN = OPRM1 PE = 1 SV = 2	0.070283	0.724809
P0DJI8	30	19	189.2047	0.000380854	5.2211	CRP >30	CRP < 30	Serum amyloid A-1 protein OS = Homo sapiens GN = SAA1 PE = 1 SV = 1	0.253761	0.705353
P02655	9	8	116.1691	0.000684848	3.4281	CRP < 30	CRP >30	Apolipoprotein C-II OS = Homo sapiens GN = APOC2 PE = 1 SV = 1	-0.118406	-0.715632
Q96PD5	51	46	510.5228	0.000633028	1.5371	CRP < 30	CRP >30	N-acetylmuramoyl-L-alanine amidase OS = Homo sapiens GN = PGLYRP2 PE = 1 SV = 1	-0.291687	-0.729485

**Fig 2:**
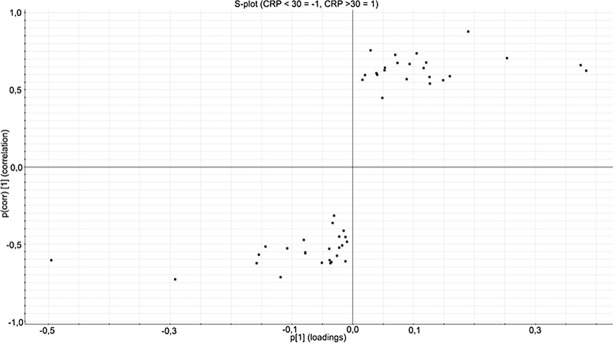
S-plot of the CRC samples with CRP <30 and >30 obtained from the OPLS-DA analysis. Only proteins with an ANOVA p-value of less than 0.05 are shown. The S-plot shows the relationship between the correlation (p(corr)) and the covariance (p), where variables having a p(corr) value higher than 0.7 or lower than -0.7 are considered significantly different. Data are log10-transformed and mean-centered. The proteins in the upper right section of the plot are downregulated and the proteins in the lower left section upregulated in the CRP >30 category as compared to the CRP <30 category.

The proteins passing the cut-off from the samples divided into CRP <30 or >30 are shown in Fig 2, visualized on an S-plot.

#### Pathway analysis

**Fig 3:**
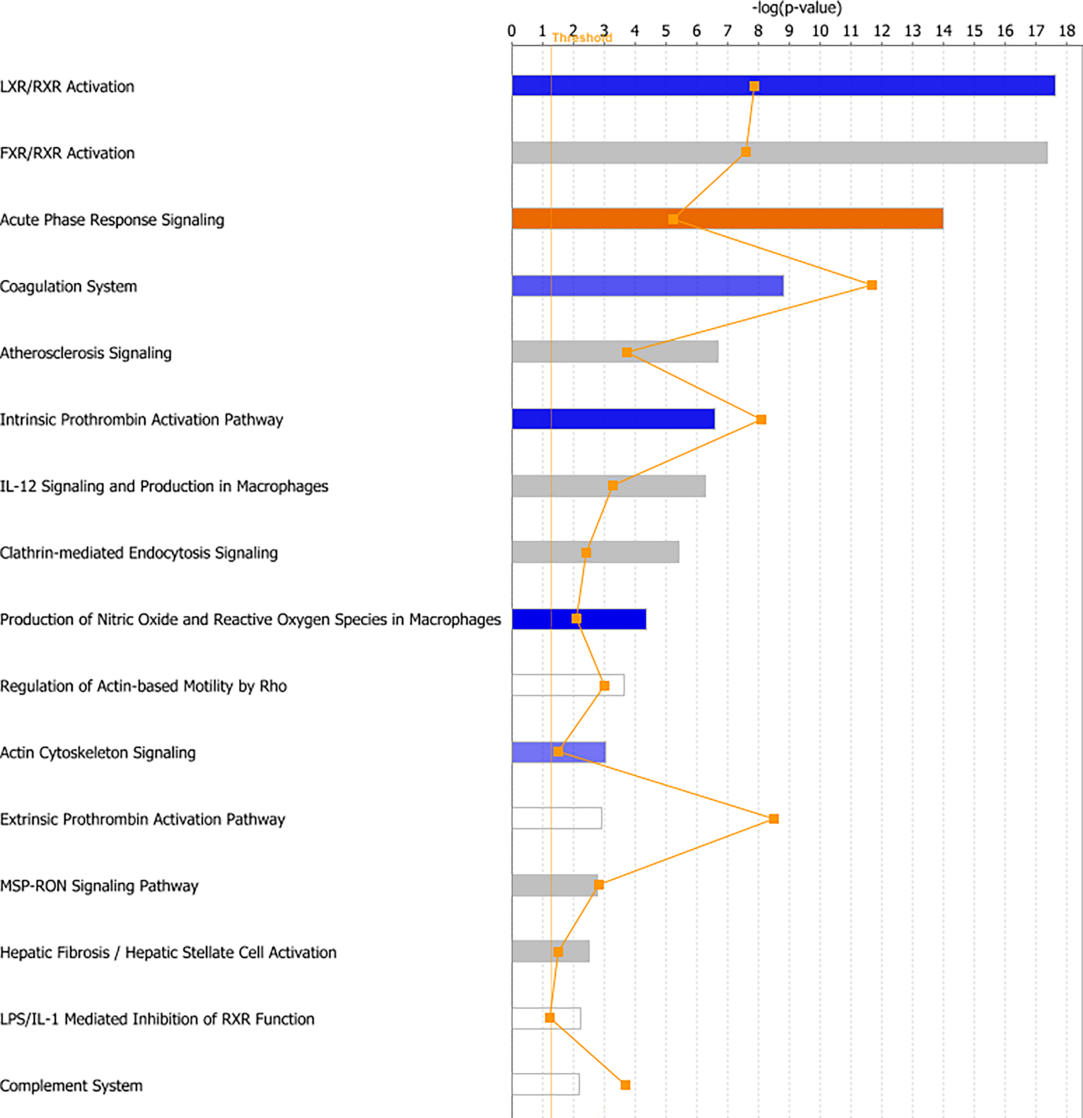
Canonical pathways found by core analysis in IPA of the CRP categories. The top canonical pathways enriched by Ingenuity Pathway Analysis (IPA) are shown above. The straight orange vertical line running through the bars is the threshold for the p-value for the particular pathway’s enrichment. The horizontal axis is the–log(p-value) and the vertical axis shows the given pathways.

In this study, we used two tools for pathway analysis: Integrated Molecular Pathway Level Analysis (IMPaLa) and Ingenuity Pathway Analysis (IPA). IMPaLa was used for pathway over-representation analysis and the results when data were analyzed between categories CRP <30 and >30 are given in S4 Table. Complement and coagulation cascades, as well as vitamin B12 metabolism and the selenium micronutrient network were found to be enriched. We also performed pathway analysis using IPA and found multiple canonical pathways, molecular and cellular functions, as well as networks that were enriched in the CRP >30 dataset. Among the canonical pathways that were enriched were LXR/RXR activation, FXR/RXR activation, acute phase response signaling and, similar to the results from the IMPaLa analysis, the complement system. Some of the top canonical pathways are shown in Fig 3.

**Fig 4:**
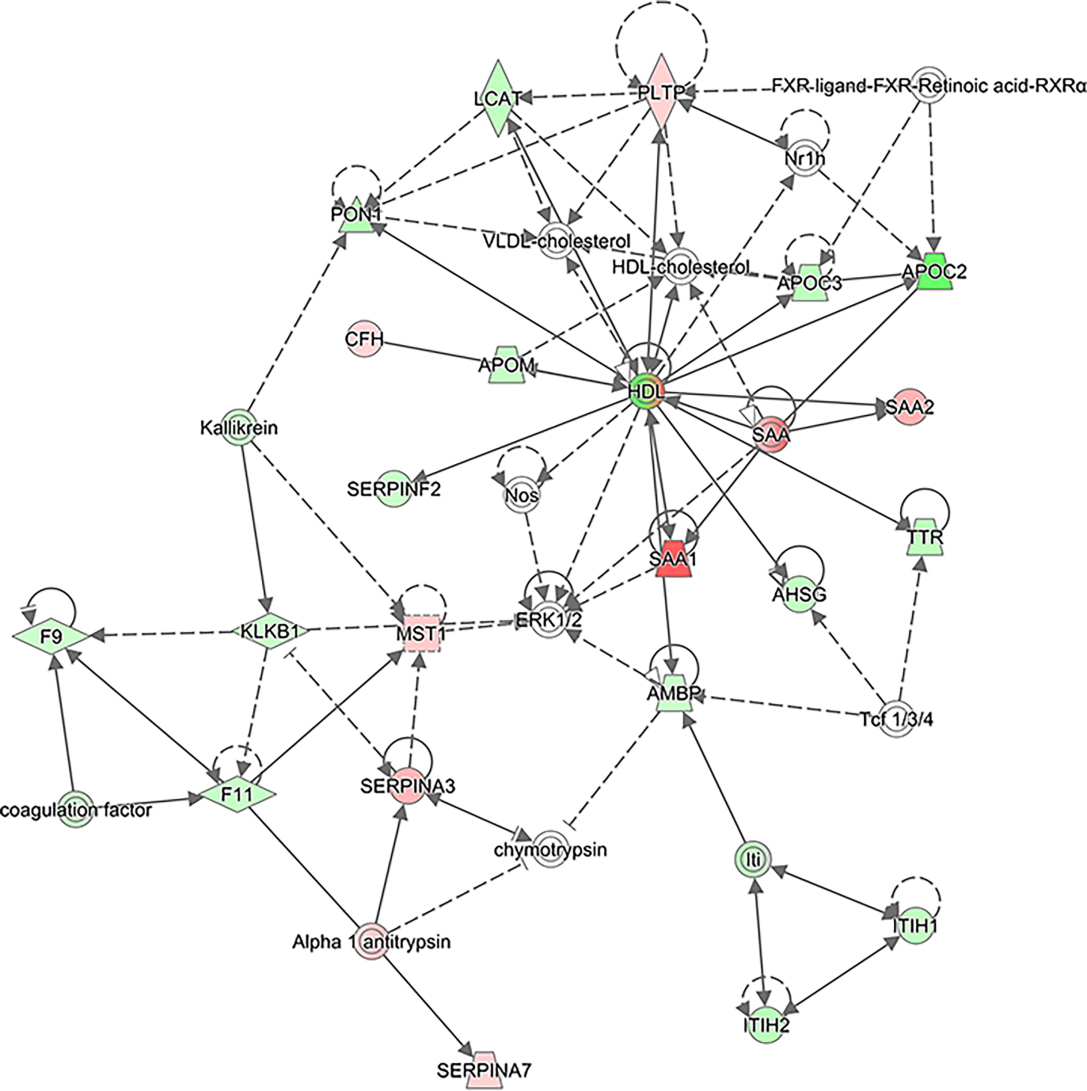
The top network of proteins found to be up- or downregulated by IPA when analyzed according to the CRP categories. Only proteins passing the cut-off of 0.05 for ANOVA were used. Upregulated proteins are shown in red and downregulated proteins in green. White proteins are proteins involved in these pathways but not detected in our dataset.

IPA also revealed protein networks enriched in the CRP >30 category when compared to the CRP <30 category, and the top network is presented in Fig 4. As can be seen, several serum amyloid A (SAA) proteins such as SAA1 and SAA2 are upregulated here. Several members of the serpin family, SERPINA3, alpha 1 antitrypsin (encoded by the SERPINA1 gene) and SERPINA7, are also upregulated in this network. The top six networks found by IPA analysis and the full list of proteins involved in these networks are given in S5 Table.

### Survival dataset

The serum samples used in this study were also analyzed by comparing long and short 5-year survival categories. When data were analyzed according to 5-year survival, 19 proteins passed the cut-off of ANOVA p-value 0.05 when the highest mean was set to long and 12 proteins when the highest mean was set to short. The first 20 proteins to pass the cut-off are presented in Table 3, and all 31 proteins are listed in S6 Table. Again, our main criterion for identifying proteins different between the classes of samples was ANOVA p-values and therefore, proteins with p-values greater than 0.05 were not considered to be different.

**Table 3:** The first 20 proteins with 2 or more unique peptides analyzed according to 5-year survival and passing the cut-off of 0.05 for ANOVA. Accession, peptide count, unique peptides, confidence score, ANOVA p-value, maximum fold change and highest and lowest mean condition as well as the full protein name are given in the table.

Accession	Peptide count	Unique peptides	Confidence score	Anova (p)	Max fold change	Highest mean condition	Lowest mean condition	Description
P02750	42	34	358.9	0.0019	2.23	Survival < 5 a	Survival > 5 a	Leucine-rich alpha-2-glycoprotein OS = Homo sapiens GN = LRG1 PE = 1 SV = 2
P00747	148	118	1396.1	0.004178	1.28	Survival > 5 a	Survival < 5 a	Plasminogen OS = Homo sapiens GN = PLG PE = 1 SV = 2
P02656	24	21	150.2	0.0049	1.48	Survival > 5 a	Survival < 5 a	Apolipoprotein C-III OS = Homo sapiens GN = APOC3 PE = 1 SV = 1
P05452	23	17	245.4	0.0050	1.51	Survival > 5 a	Survival < 5 a	Tetranectin OS = Homo sapiens GN = CLEC3B PE = 1 SV = 3
P42081	3	2	15.3	0.0089	1.52	Survival > 5 a	Survival < 5 a	T-lymphocyte activation antigen CD86 OS = Homo sapiens GN = CD86 PE = 1 SV = 2
Q6ZS52	2	2	10.5	0.0109	2.05	Survival < 5 a	Survival > 5 a	Putative uncharacterized protein FLJ45825 OS = Homo sapiens PE = 5 SV = 1
P78380	2	2	9.3	0.0115	3.31	Survival < 5 a	Survival > 5 a	Oxidized low-density lipoprotein receptor 1 OS = Homo sapiens GN = OLR1 PE = 1 SV = 1
Q96BN8	3	2	13.4	0.0129	1.70	Survival > 5 a	Survival < 5 a	Ubiquitin thioesterase otulin OS = Homo sapiens GN = OTULIN PE = 1 SV = 3
O14986	7	7	48.0	0.0155	1.48	Survival > 5 a	Survival < 5 a	Phosphatidylinositol 4-phosphate 5-kinase type-1 beta OS = Homo sapiens GN = PIP5K1B PE = 1 SV = 2
P49908	13	8	95.9	0.0186	1.32	Survival > 5 a	Survival < 5 a	Selenoprotein P OS = Homo sapiens GN = SEPP1 PE = 1 SV = 3
P00734	103	78	785.3	0.0209	1.19	Survival > 5 a	Survival < 5 a	Prothrombin OS = Homo sapiens GN = F2 PE = 1 SV = 2
P02655	9	8	116.2	0.0214	2.25	Survival > 5 a	Survival < 5 a	Apolipoprotein C-II OS = Homo sapiens GN = APOC2 PE = 1 SV = 1
P35527	15	13	108.1	0.0225	1.67	Survival < 5 a	Survival > 5 a	Keratin. type I cytoskeletal 9 OS = Homo sapiens GN = KRT9 PE = 1 SV = 3
P02741	3	2	29.3	0.0233	2.08	Survival < 5 a	Survival > 5 a	C-reactive protein OS = Homo sapiens GN = CRP PE = 1 SV = 1
P15169	26	21	248.7	0.0235	1.51	Survival < 5 a	Survival > 5 a	Carboxypeptidase N catalytic chain OS = Homo sapiens GN = CPN1 PE = 1 SV = 1
P01042	68	58	697.7	0.0275	1.11	Survival > 5 a	Survival < 5 a	Kininogen-1 OS = Homo sapiens GN = KNG1 PE = 1 SV = 2
P24385	2	2	18.9	0.0302	1.47	Survival > 5 a	Survival < 5 a	G1/S-specific cyclin-D1 OS = Homo sapiens GN = CCND1 PE = 1 SV = 1
P29622	2	2	13.3	0.0324	1.77	Survival > 5 a	Survival < 5 a	Kallistatin OS = Homo sapiens GN = SERPINA4 PE = 1 SV = 3
Q02410	4	2	18.5	0.0330	1.68	Survival > 5 a	Survival < 5 a	Amyloid beta A4 precursor protein-binding family A member 1 OS = Homo sapiens GN = APBA1 PE = 1 SV = 3
P10909	61	45	556.9	0.0345	1.19	Survival > 5 a	Survival < 5 a	Clusterin OS = Homo sapiens GN = CLU PE = 1 SV = 1

#### PCA

**Fig 5:**
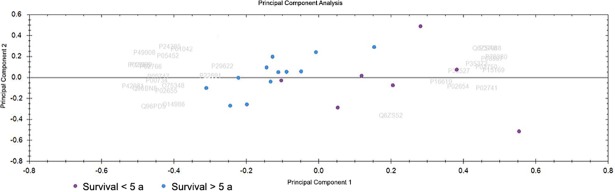
PCA where blue dots are CRC samples with a long 5-year survival and purple dots are CRC samples with a short 5-year survival. The PCA was performed on proteins with 2 or more unique peptides that passed the cut-off of 0.05 for ANOVA.

The PCA biplot showing long and short 5-year survival samples is shown in Fig 5. Only proteins with 2 or more unique peptides and an ANOVA p-value of less than 0.05 were considered for this PCA. A separation between the two groups can be seen here, with very few samples overlapping slightly.

#### OPLS-DA

An S-plot was also generated for the proteins found in the analysis of long and short 5-year survival. Again, proteins passing the cut-off value of +0.7 or -0.7 for p(corr) values were considered to be significantly different and are presented in Table 4. Apolipoprotein C-I (APOC1), Oxidized low-density lipoprotein receptor 1 (OLR1) and CRP were found to be upregulated in the short 5-year survival category, while tetranectin and V-type proton ATPase subunit G 1 (ATP6V1G1) were found to be downregulated.

**Table 4:** Proteins significantly different in the S-plot generated by comparing long and short 5-year survival. Peptide count is the total number of peptides found for the given protein and unique peptides are the number of peptides unique to that protein out of the total peptides. Confidence score, ANOVA p-value, highest and lowest mean condition, the full name of the protein, covariance (p[1]) and correlation (p(corr)[1]) are shown in the table.

Primary accession	Peptide count	Unique peptides	Confidence score	Anova (p)	Max fold change	Highest mean condition	Lowest mean condition	Description	p[1]	p(corr)[1]
P02654	9	5	60.50	0.0345	2.0448	SHORT	LONG	Apolipoprotein C-I OS = Homo sapiens GN = APOC1 PE = 1 SV = 1	0.0528039	0.759156
Q6ZSA8	2	2	16.43	0.0484	2.5360	SHORT	LONG	Putative uncharacterized protein FLJ45684 OS = Homo sapiens PE = 5 SV = 2	0.12572	0.729375
P78380	2	2	9.25	0.0115	3.3056	SHORT	LONG	Oxidized low-density lipoprotein receptor 1 OS = Homo sapiens GN = OLR1 PE = 1 SV = 1	0.122362	0.720305
P02741	3	2	29.30	0.0233	2.0843	SHORT	LONG	C-reactive protein OS = Homo sapiens GN = CRP PE = 1 SV = 1	0.0936024	0.712781
P05452	23	17	245.35	0.0050	1.5138	LONG	SHORT	Tetranectin OS = Homo sapiens GN = CLEC3B PE = 1 SV = 3	-0.100494	-0.734962
O75348	3	2	21.63	0.0486	1.4632	LONG	SHORT	V-type proton ATPase subunit G 1 OS = Homo sapiens GN = ATP6V1G1 PE = 1 SV = 3	-0.0176084	-0.737745

**Fig 6:**
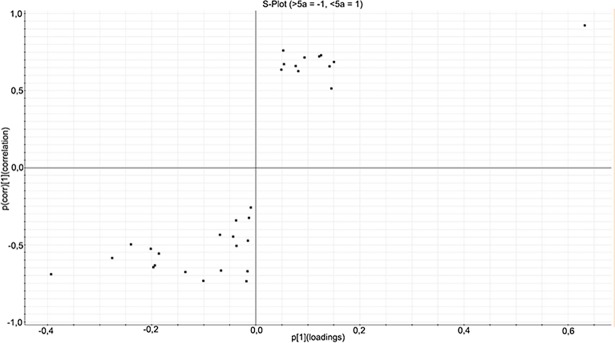
S-plot of the CRC samples with long or short 5-year survival obtained from the OPLS-DA analysis. Only proteins with an ANOVA p-value of less than 0.05 are shown. Variables having a p(corr) value higher than 0.7 or lower than -0.7 are considered significantly different. Data are log10-transformed and mean-centered. The proteins in the upper right section of the plot are downregulated and the proteins in the lower left section upregulated in the short 5-year survival category as compared to the long 5-year survival category.

The proteins passing the cut-off from the samples divided into long and short 5-year survival are shown in Fig 6, visualized on an S-plot.

#### Pathway analysis

**Fig 7:**
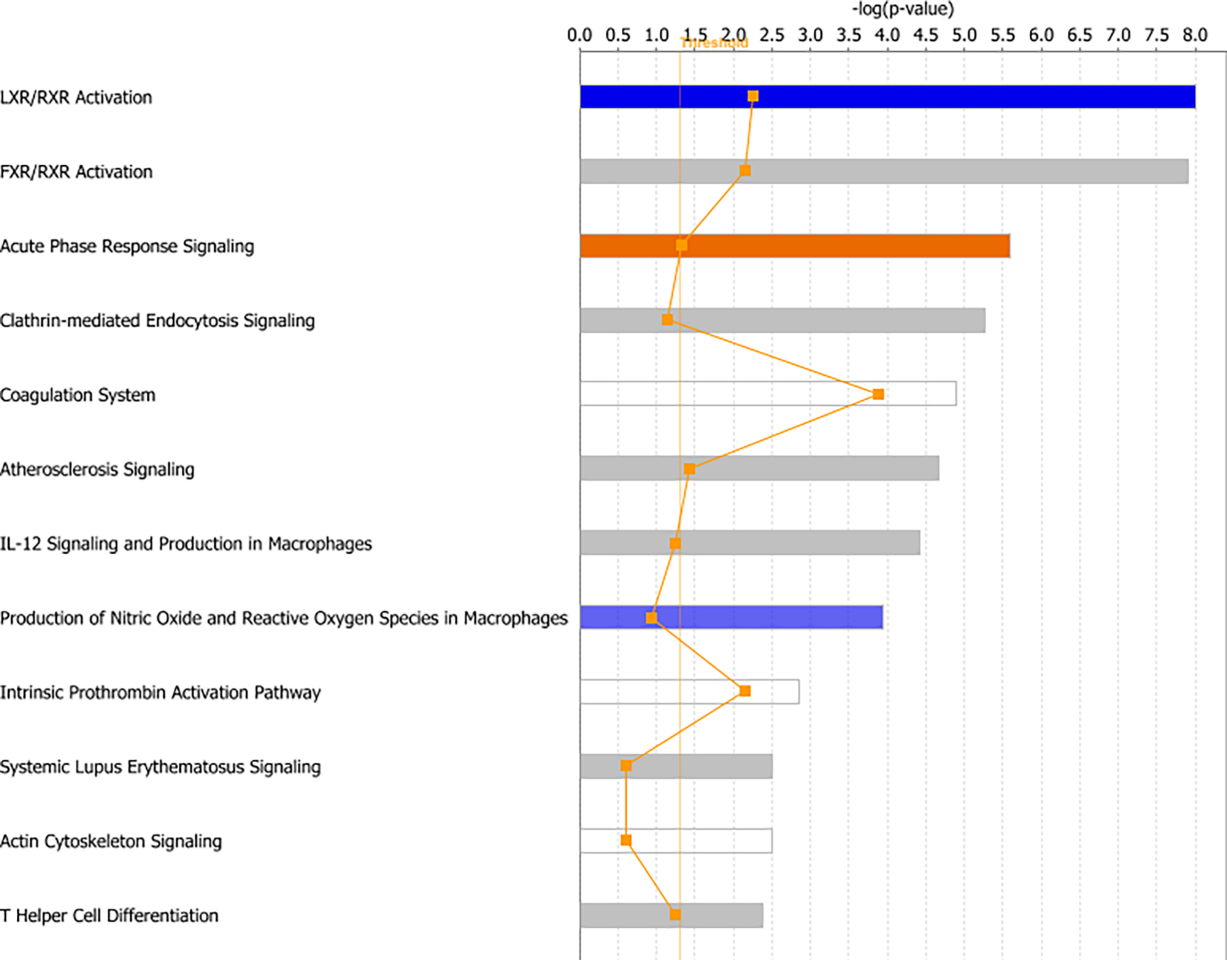
Canonical pathways found by core analysis in IPA of the 5-year survival categories. The top canonical pathways enriched by Ingenuity Pathway Analysis (IPA) are shown above. The straight orange vertical line running through the bars is the threshold for the p-value for the particular pathway’s enrichment. The horizontal axis is the–log(p-value) and the vertical axis shows the given pathways.

IMPaLa was again used for pathway over-representation analysis and the results when data were analyzed according to long and short 5-year survival are given in S7 Table. Signaling cascades involving platelet degranulation, activation, signaling and aggregation were found to be enriched. Complement and coagulation cascades and the statin pathway were also enriched. We also performed pathway analysis using IPA on this dataset and found multiple canonical pathways, molecular and cellular functions, as well as networks that were enriched in the short 5-year survival dataset. The canonical pathways enriched were similar to those found in the CRP >30 dataset, such as LXR/RXR activation, FXR/RXR activation and acute phase response signaling. Some of the top canonical pathways are shown in Fig 7.

**Fig 8:**
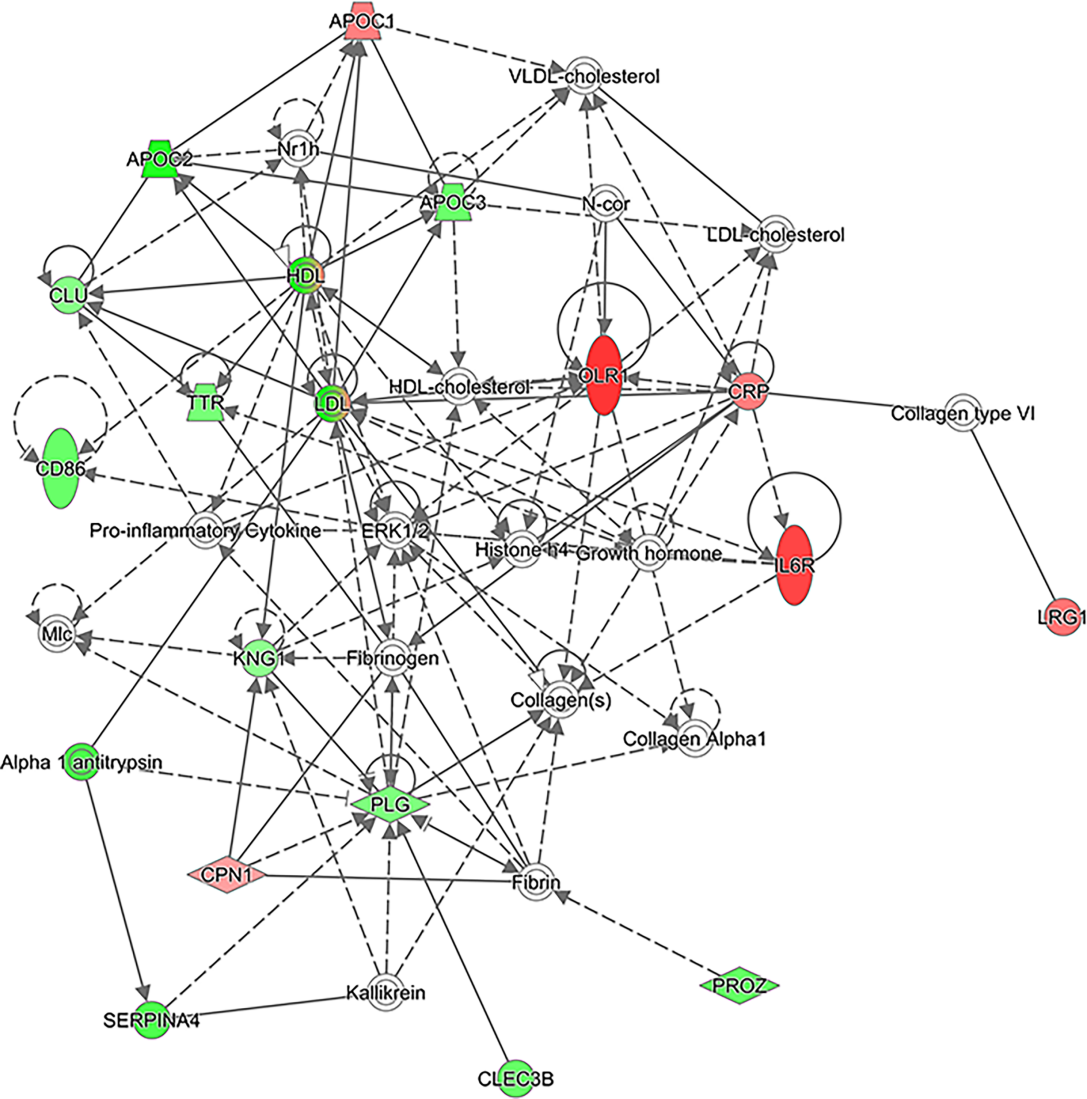
The top network of proteins found to be up- or downregulated by IPA when analyzed according to the survival categories. Only proteins passing the cut-off of 0.05 for ANOVA were used. Upregulated proteins are shown in red and downregulated proteins in green. White proteins are proteins involved in these pathways but not detected in our dataset.

IPA also revealed protein networks enriched in the short 5-year survival category when compared to the long 5-year survival category, and the top network is presented in Fig 8. The top four networks found by IPA analysis and the full list of proteins involved in these networks are given in S8 Table. As can be seen, high-density lipoprotein (HDL) and low-density lipoprotein (LDL) are downregulated here, while OLR1 and CRP are upregulated. Several apolipoproteins were also involved in this network: APOC1 is upregulated while APOC2 and APOC3 are downregulated in the short 5-year survival category as compared to the long 5-year survival category.

## Discussion

In recent years, the incidence of CRC has only significantly decreased in the United States, while both incidence and death rates are rapidly increasing in other, less-developed countries [1, 3]. CEA is the most widely used biomarker in CRC but is of little help in detecting early CRC, which limits its usefulness. Due to a lack of effective, non-invasive biomarkers, new biomarkers are needed to efficiently diagnose and screen for CRC [11].

In this study, we retrospectively recruited 19 CRC patients and studied preoperative serum samples by UPLC-UDMS^E^. We quantified 256 proteins with 2 or more unique peptides, which were used for analysis. An elevated concentration of CRP is linked to poor prognosis in CRC patients [14], and we therefore decided to analyze data according to CRP values to find differentially expressed proteins. We also analyzed the same samples according to long and short 5-year survival in order to discover proteins that could potentially be of use in predicting outcome.

By using proteomic methods, serum proteins can be studied to discover new potential biomarkers. The discovery of a biomarker that could be measured from blood samples is ideal due to the minimal invasiveness and ease of obtaining such samples. Abundant proteins such as albumin represent more than 99% of the proteins found in serum. This complicates the discovery of novel protein biomarkers, and the abundant proteins were removed to reduce the complexity of serum samples. This enables the discovery of low-abundance proteins, some of which may be of clinical importance [21, 22].

PCA analysis of the samples divided into categories according to CRP (CRP <30 and >30) and 5-year survival (short and long), showed a separation on the PCA biplot. We continued with OPLS-DA modeling and generation of an S-plot. Proteins passing the cut-off value of +0.7 or -0.7 for p(corr) values were considered to be significantly different (Table 2 and Table 4). Among the proteins upregulated in the CRP >30 category are IGFBP2 and SAA1 (also abbreviated SAA).

IGFBP2 is one of the six binding proteins that modulate the interactions of Insulin-like growth factors (IGFs) with their receptor. IGFs have important roles in growth and cell proliferation [23]. IGFBP2 has been identified as a potential biomarker for CRC in previous studies and elevated levels of IGFBP2 have been shown to be associated with poor survival in CRC patients [24, 25]. Elevated serum levels of IGFBP2 have also been discovered in patients with breast [26], ovarian [27] and prostate cancer [28]. Our findings further support the role of IGFBP2 as a biomarker for CRC, although further validation is required.

SAA is a lipoprotein whose concentration increases during the acute phase of the inflammatory response (APR) [29]. SAA has been proposed as a biomarker for CRC, albeit in a small study [30]. Elevated serum levels of SAA have also been identified in patients with gastric [31], lung [32], nasopharyngeal [33], renal cell [34] and endometrial cancer [35], where they correlated with poor prognosis. In our study, we also found that PCDHA11 and OPRM1 were upregulated in the CRP >30 category when compared to the CRP <30 category. These proteins are, to the best of our knowledge, identified here as potential biomarkers for the first time.

Pathway analysis by IPA and IMPaLa mostly found pathways involved in lipid metabolism, as well as complement and coagulation cascades and acute phase response signaling to be enriched, implying that these are the main perturbed pathways in CRC patients with high CRP. IPA pathway analysis showed LXR/RXR activation and FXR/RXR activation as the most enriched pathways (Fig 3). LXR/RXR activation is suggested to have a role in the absorption of cholesterol, and LXRs also regulate the biliary excretion of cholesterol [36, 37]. Farnesoid X receptors (FXRs) also form heterodimers with RXRs, and FXR/RXR activation plays a role in the regulation of both cholesterol and bile acid metabolism [38, 39]. Deregulation of lipid metabolism has increasingly been recognized as a feature of cancer cells [40, 41]. SAA has a role in lipid metabolism, linking the results of the IPA pathway analysis to one of the proteins found by OPLS-DA and S-plot analysis.

Enhanced levels of coagulation markers have been shown to occur in CRC patients and advanced cancer is also associated with a hypercoagulable state, which is in line with our findings of enriched coagulation cascades in pathway analysis [42, 43]. The enrichment of complement cascades and acute phase response signaling in the CRP >30 category is logical, due to the presence of an inflammatory response in these patients.

The same analyses were performed for the short and long 5-year survival categories. Among the proteins upregulated in the short 5-year survival category are APOC1, OLR1 and CRP. APOC1 is a lipoprotein that, among other functions, helps to maintain HDL structure [44]. High preoperative serum levels of APOC1 have been shown to correlate with poor prognosis in pancreatic cancer patients [45]. Serum APOC1 needs to be further investigated to evaluate its use as a biomarker, especially in CRC, for which there are no studies. OLR1, a receptor for low-density lipoproteins, has been proposed to function as an oncogene [46], although the role of OLR1 in CRC is unknown. CRP was also upregulated, which was to be expected, as we deliberately included patients with elevated CRP levels in this study.

Pathway analysis by IPA found similar pathways to be enriched in the category with short 5-year survival as in the category with high CRP. Pathway analysis by IMPaLa showed pathways involving platelets as well as complement and coagulation cascades to be enriched. Platelets have long been implicated in the spread of cancer, and cancer patients often have a high platelet count and turnover. An elevated platelet count has also been shown to be indicative of poor prognosis in CRC patients [47, 48].

In this study, we analyzed samples according to CRP (<30 vs. >30) and 5-year survival (short vs. long) to identify differences in serum proteins within these categories and to find proteins that are linked to patient outcome and prognosis. These proteins could be used to select patients with a poor prognosis that would benefit from a more aggressive treatment regimen and help those with a good prognosis to avoid it. Here, we have identified multiple potential biomarkers, although they require further validation.

This study was limited due to its small size, as only 19 patients were included in this study. CRC is a heterogeneous disease that can be divided into several subtypes characterized by distinct molecular pathologies and clinical features. For example, microsatellite instability (MSI) is associated with CRC prognosis, with MSI-high CRC showing better survival than microsatellite stable (MSS) CRC [49, 50]. Lack of tumor molecular data for our dataset therefore leads to some additional limitations of this study. Due to the small size of this study, it was not feasible to subdivide our material into subcategories: for example, only around 15% of CRCs are MSI-high [49], giving us very few patients with MSI-high CRC that could be used for comparisons.

Another limitation of the study could be that multiple hypothesis testing correction was not employed. We realize that it might lead to a few results being falsely significant, but it has to be considered that multiple testing correction is often over-stringent, and significant inferences are usually missed in trying to control a Type I error. This is called committing a Type II error and it’s always the trade-off between Type I and II errors that a researcher has to decide between. Moreover, to establish a useful prognostic biomarker, one usually starts with a number of lead candidates, and at the validation stage, in a very large cohort, multiple hypothesis correction can be employed. Also, popular methods of multiple testing corrections, such as Bonferroni and Sidak methods, are under-powered when variable measurements are correlated [51], which is often the case with proteomic measurements. Some of the proteins we identified here have been recognized as potential biomarkers previously, whereas some have been identified as potential biomarkers for the first time, to the best of our knowledge. This pilot study will therefore pave the way for further studies, with the aim to provide better biomarkers for CRC patients.

## Supporting information

S1 Table Colorectal cancer patients included in this study. The table shows patients’ gender, age, stage and location of cancer, preoperative CRP value, survival, and cause of death or alive.
(XLSX)

S2 Table All 256 proteins quantified with two or more unique peptides. Accession, peptide count, unique peptides, confidence score, ANOVA p-value, maximum fold change and highest and lowest mean condition as well as the full protein name are given in the table.
(XLSX)

S3 Table All 71 proteins with 2 or more unique peptides analyzed according to CRP and passing the cut-off of 0.05 for ANOVA. Accession, peptide count, unique peptides, confidence score, ANOVA p-value, maximum fold change and highest and lowest mean condition as well as the full protein name are given in the table.
(XLSX)

S4 Table All 31 proteins with 2 or more unique peptides analyzed according to 5-year survival and passing the cut-off of 0.05 for ANOVA. Accession, peptide count, unique peptides, confidence score, ANOVA p-value, maximum fold change and highest and lowest mean condition as well as the full protein name are given in the table.
(XLSX)

S5 Table IMPaLa pathway over-representation analysis results of the CRP categories are shown here. Pathway name, source, number of overlapping genes, number of all pathway genes and P and Q values of enrichment are listed.
(XLSX)

S6 Table This table shows the proteins in and the function of the six top networks found by IPA analysis of the CRP categories. Only proteins passing the cut-off of 0.05 for ANOVA were used.
(XLS)

S7 Table IMPaLa pathway over-representation analysis results of the 5-year survival categories are shown here. Pathway name, source, number of overlapping genes, number of all pathway genes and P and Q values of enrichment are listed.
(XLSX)

S8 Table This table shows the proteins in and the function of the four top networks found by IPA analysis of the 5-year survival categories. Only proteins passing the cut-off of 0.05 for ANOVA were used.
(XLS)
